# Attenuated mutants of *Salmonella enterica* Typhimurium mediate melanoma regression via an immune response

**DOI:** 10.3389/ebm.2024.10081

**Published:** 2024-06-21

**Authors:** Genesy Pérez Jorge, Marco Gontijo, Marina Flóro e Silva, Isabella Carolina Rodrigues Dos Santos Goes, Yessica Paola Jaimes-Florez, Lilian de Oliveira Coser, Francisca Janaína Soares Rocha, Selma Giorgio, Marcelo Brocchi

**Affiliations:** ^1^ Departamento de Genética, Evolução, Microbiologia e Immunologia, Instituto de Biologia, Universidade Estadual de Campinas—UNICAMP, Campinas, SP, Brazil; ^2^ Research Group: Statistics and Mathematical Modeling Applied to Educational Quality, University of Sucre, Sincelejo, Sucre, Colombia; ^3^ Department of Molecular Genetics and Microbiology, Duke University Medical Center, Duke Medicine Cir, Durham, NC, United States; ^4^ Departamento de Biologia Animal, Instituto de Biologia, Universidade Estadual de Campinas, Campinas—UNICAMP, Campinas, SP, Brazil; ^5^ GIMBIO Group, Department of Microbiology, Faculty of Basic Sciences, Universidad de Pamplona, Pamplona, Colombia; ^6^ Departamento de Biologia Estrutural e Funcional, Laboratório de Regeneração Nervosa, Instituto de Biologia, Universidade Estadual de Campinas—UNICAMP, Campinas, SP, Brazil; ^7^ Área Acadêmica de Medicina Tropical, Centro de Ciências Médicas, Universidade Federal de Pernambuco, Recife, Pernambuco, Brazil

**Keywords:** *S. enterica* Typhimurium, melanoma, mutants, anticancer, macrophage

## Abstract

The lack of effective treatment options for an increasing number of cancer cases highlights the need for new anticancer therapeutic strategies. Immunotherapy mediated by *Salmonella enterica* Typhimurium is a promising anticancer treatment. Candidate strains for anticancer therapy must be attenuated while retaining their antitumor activity. Here, we investigated the attenuation and antitumor efficacy of two *S. enterica* Typhimurium mutants, Δ*tolRA* and Δ*ihfABpmi*, in a murine melanoma model. Results showed high attenuation of Δ*tolRA* in the *Galleria mellonella* model, and invasion and survival in tumor cells. However, it showed weak antitumor effects *in vitro* and *in vivo*. Contrastingly, lower attenuation of the attenuated Δ*ihfABpmi* strain resulted in regression of tumor mass in all mice, approximately 6 days after the first treatment. The therapeutic response induced by Δ*ihfABpmi* was accompanied with macrophage accumulation of antitumor phenotype (M1) and significant increase in the mRNAs of proinflammatory mediators (TNF-α, IL-6, and iNOS) and an apoptosis inducer (Bax). Our findings indicate that the attenuated Δ*ihfABpmi* exerts its antitumor activity by inducing macrophage infiltration or reprogramming the immunosuppressed tumor microenvironment to an activated state, suggesting that attenuated *S. enterica* Typhimurium strains based on nucleoid-associated protein genes deletion could be immunotherapeutic against cancer.

## Graphical Abstract

**Figure:**
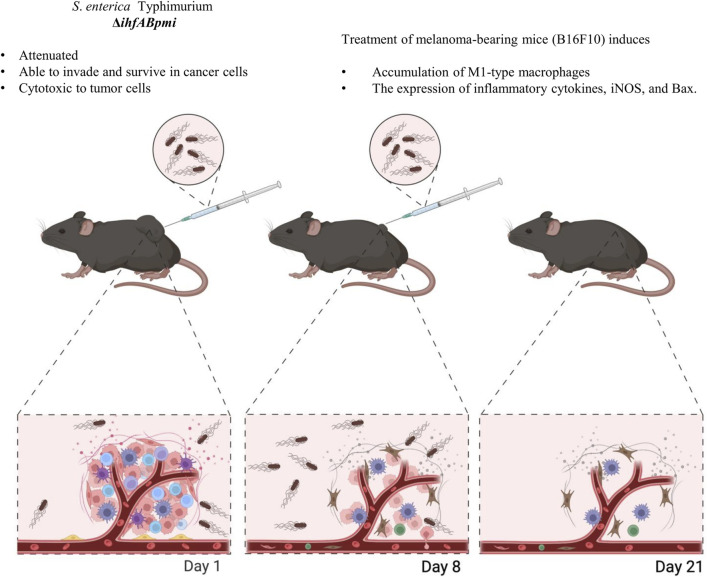
Created with BioRender.com.

## Impact statement

Melanoma is the most common form of malignancy in Caucasians globally. Recent advances in cancer treatment are still insufficient to mitigate mortality and recurrence rates, primarily due to tumor heterogeneity. Live tumor-targeting bacteria represent a unique therapeutic option to overcome cancer therapeutics’ high toxicity, drug penetration, and resistance. Here, we show that live attenuated *Salmonella enterica* Typhimurium mutants have antitumor potential *in vivo* against the aggressive melanoma model with no observable side effects. Intratumoral injection of attenuated *S. enterica* Typhimurium mediated the reduction of the tumor mass. Furthermore, we demonstrate that the tumor regression caused by our triple mutant is related to a shift to a pro-inflammatory response. These findings delineate new mutants in the fight against cancer and possible mechanisms of localized bacteria-mediate inflammation leading to mouse tumor regression. Advances in our understanding of bacteria-mediated cancer treatment will help guide the development of new and improved cancer treatment therapies.

## Introduction

Cancer is a malignant disease difficult to treat efficiently, resulting in significant global, social, and economic burden [1]. Despite major advances in cancer treatment, including the development of surgery, chemotherapy, radiotherapy, and antibody immunotherapy, mortality and recurrence rates remain high due to the complexity of the disease and limitations of currently available treatment options [1–3]. Surgical resection of tumors with surrounding healthy tissue represents the primary and most favorable treatment option for patients with early-stage cancer (when the tumor is small and localized). However, due to the silent nature of oncogenesis, most patients are often diagnosed at advanced stages of the disease when surgical resection is not a viable treatment option. The standard anticancer treatments of chemo- and radiotherapy have low specificity and high toxicity, resulting in severe side effects [4]. Therapies based on monoclonal antibodies also show limited immune response and penetration, with only a minority of patients that respond to treatment [5]. Therefore, new therapeutic approaches for efficient treatment of cancer patients are urgently needed. In this context, *Salmonella enterica* Typhimurium–mediated immunotherapy represents a viable solution for cancer treatment.

*Salmonella enterica* Typhimurium strains have been extensively explored in cancer immunotherapy due to several innate characteristics, including the targeting of intrinsic tumors, penetration of hypoxic areas [6, 7] and intrinsic antitumor activity [8]. The antitumor activity of *S. enterica* Typhimurium is mainly attributed to activation of the immune system [8–12]. Infection by *S. enterica* Typhimurium in the tumor microenvironment alerts the immune system, activating oncolytic mechanisms such as increased expression of interferon γ, inducible nitric oxide synthase (iNOS), interleukin-1β (IL-1β), and tumor necrosis factor α (TNF-α) [10], as well as decreased levels of transforming growth factor-β (TGF-β), vascular endothelial growth factor (VEGF), and anti-inflammatory cytokines. Moreover, *S. enterica* Typhimurium can promote the recruitment of immune cells, such as dendritic cells, neutrophils, lymphocytes, and macrophages [10–13]. Taken together, these mechanisms alter the immunosuppressed antitumor microenvironment into an immunoactive environment that promotes tumor cell destruction.

Macrophage infiltration and phenotype after *S. enterica* administration in the tumor microenvironment has been little studied [13]. Alternately activated macrophages or M2-type macrophages promote immunosuppression and are characterized by decreased iNOS and TNF-α expression, and positively influence tumor growth, metastases, angiogenesis, and extracellular matrix remodeling, while classically activated or M1-type macrophages exhibit phagocytic activity, can promote a Th1 response, and are associated with tumor growth suppression [10, 14, 15]. Lipopolysaccharide (LPS) can induce phenotypic changes in macrophages [15, 16]. Previously, it was shown that *S. enterica* Typhimurium mutants could induce macrophage infiltration and reprogram the M2 to M1 macrophage polarization [10, 11, 16], suggesting their potential use to combat tumor progression.

Different strains of *S. enterica* Typhimurium have shown promising results *in vitro* and *in vivo* [17–22]. However, strains evaluated in clinical trials have not shown antitumor efficacy [23–25], making it essential to explore new strains of *S. enterica* Typhimurium with antitumor potential. A good anticancer therapy candidate must have a balance between attenuation and antitumor activity. Wild-type *S. enterica* Typhimurium strains are unsuitable for cancer therapy because they can cause severe sepsis in the host. Highly attenuated strains are inefficient because they cannot stimulate the immune system for tumor elimination [26].

Considering the need to develop safe strains with antitumor efficacy, in this study, we constructed two mutants of *S. enterica* Typhimurium and evaluated their therapeutic efficacy, as well the induction of macrophage infiltration and production of inflammatory mediators in tumors. The Δ*tolRA* mutant strain lacks two cell envelope proteins, and the Δ*ihfABpmi* mutant is deficient in 6-phosphomannose isomerase and integration host factor (IHF), a nucleoid-associated protein. Both mutants showed attenuation of virulence and antitumor potential in a murine melanoma model. Furthermore, the antitumor efficacy of the Δ*ihfABpmi* mutant was associated with the accumulation of phenotype M1 macrophages in melanoma tumors.

## Materials and methods

### Bacterial strains, plasmids, primers, and culture media

The bacterial strains, plasmids, and primers used in this study are described in Supplementary Table S1. Bacteria were grown aerobically at 37°C on LB agar, LB broth (prepared according to Sambrook and Russell, 2001 [27]), MacConkey (Kasvi, Brazil), and Salmonella-Shigella (SS) media (Oxoid, United Kingdom). Ampicillin (100 μg/mL; Sigma, Spain), Kanamycin (50 μg/mL; Sigma, Spain), chloramphenicol (25 μg/mL; USB, United Kingdom), or mannose (0.5%, Sigma, Spain) were used, as required. The bacterial strains used in this study were stored at −80°C in 20% glycerol in an LB medium.

### *In vitro* growing conditions

Bacteria were seeded on LB agar and incubated at 37°C for 16–18 h. Colonies grown on LB agar were inoculated into LB broth and cultured for 16–18 h at 37°C under agitation (150 rpm). The next day, the culture was diluted 1:100 in fresh LB broth and grown to the exponential phase (∼10^8^ CFU/mL) under the above conditions. The culture was centrifuged at 4,000 × g for 5 min, and the pellet was suspended in PBS. Subsequently, the bacteria were diluted to the appropriate concentration for *in vitro* and *in vivo* experiments. In all experiments, mannose was added to the culture medium (LB agar or LB broth) for the growth of the Δ*ihfABpmi* mutant.

### Construction of mutants

The new triple mutant Δ*ihfABpmi* was constructed using the λ Red recombination system followed by transduction with phage p22. We initially constructed the Δ*pmi*:*Kan* mutant by recombination-mediated allelic exchange of the *pmi* gene with the kanamycin cassette. The deletion of the *pmi* gene was verified by PCR using the detection primers described in Supplementary Table S1. Subsequently, Δ*pmi*:*Kan* was used as the donor strain for transduction with the p22 phage, and the Δ*ihfAB* mutant was used as the recipient strain, resulting in the Δ*ihfABpmi* mutant.

The double mutant Δ*tolRA* was constructed using the λ Red system as previously described [28]. We deleted the *tolR* and *tolA* genes from the ATCC 14028 chromosome in a single step, by red-mediated recombination and allelic exchange of the *tolR* and *tolA* genes with the kanamycin resistance gene. The gene deletion was confirmed by PCR using the tolRADT-F and tolRADT-R primers described in Supplementary Table S1.

### Cancer cell lineage

Cell lines B16F10 (mouse melanoma) and 5,637 (human bladder cancer) were used in this study. Cells were grown at 37°C in humidified air with 5% CO_2._ Dulbecco’s Modified Eagle medium (Thermo Fisher Scientific, Carlsbad, CA, United States) (DMEM) was used to grow B16F10 cells and RPMI 1640 (Thermo Fisher Scientific) medium was used to grow 5,637 cells. Both media were supplemented with 10% fetal bovine serum, penicillin (100 U/mL) and streptomycin (100 μg/mL) for cell growth.

### Growth curve

The *in vitro* growth of the mutants was assessed in LB broth as previously described [29]. Briefly, a 16–18 h culture was diluted (1:1,000) in LB broth and incubated at 37°C and 150 rpm. Growth was monitored for 12 h employing hourly optical density readings and CFU determination by plating on LB-agar. Three independent experiments were performed.

### Virulence in the *Galleria mellonella* model

Virulence attenuation of engineered mutants of *S. enterica* Typhimurium was evaluated *in vivo* in the *Galleria mellonella* infection model by inoculating 10 μL of a 1 × 10^6^ CFU/mL bacterial (mutant or wild-type strain) suspension in the last proleg of larvae between 200 and 250 mg. Ten larvae were used per group. Larvae survival was observed for 96 h after inoculation. The experiment was performed three times.

### LPS extraction and analysis by sodium dodecyl sulfate polyacrylamide gel electrophoresis (SDS-PAGE)

The integrity of LPS, which is associated with bacterial immunogenicity, was also analyzed by profiling LPS on a polyacrylamide gel. LPS from the mutants and wild-type strain was extracted as previously described [30]. Briefly, colonies grown on LB-agar plates were homogenized in deionized water until an OD_600_ between 0.4 and 0.5 was reached. The bacterial suspension was centrifuged at 10,000 rpm for 2 min, and the supernatant was discarded. The pellet was homogenized in a Laemmli buffer (Tris-HCl, pH 6.8 0.625 M, 2% SDS, 25% glycerol, and 0.01% bromophenol blue) incubated at 100°C for 10 min. Then, 10 µL of Proteinase K solution (2.5 mg/mL) was added, and the suspension was homogenized and incubated at 60°C for 1 h. The LPS samples were analyzed by SDS-PAGE. Polyacrylamide electrophoresis was performed in a 5% stacking gel and in a 12% separating gel, with exposure to a constant voltage of 100 V for 2 h. The separated LPS was stained with silver stain [31], by initially incubating the gel in a fixative solution (40% ethanol and 5% acetic acid) for 16 h, followed by incubation in an oxidizing solution for 10 min. After three washes in deionized water, the gel was stained for 10 min with a silver solution (0.02M NaOH, 1.5% ammonium hydroxide, and 0.7% silver nitrate), followed by three wash-steps in deionized water. Bands were developed in 0.02% formaldehyde and 0.005% acetic acid solution. After the appearance of the bands, the gel was washed in deionized water and photographed. *Pseudomonas aeruginosa* (kindly donated by Dr. Regina Lúcia Baldini—Department of Biochemistry, Instituto de Química, Universidade de São Paulo) containing the *wzz* gene deletion was used as a control for the absence of LPS. The *wzz* gene is essential for LPS biosynthesis. In *Pseudomonas aeruginosa*, strains with a deletion of this gene cannot produce LPS.

### Invasion and survival assay

The invasiveness and survival capabilities of the mutants were measured using gentamicin resistance, as previously described, with some modifications [22, 32]. Briefly, 1×10^5^ melanoma or bladder cancer cells per well were seeded in 24-well plates with antibiotic-free medium (RPMI for bladder cancer cells or DMEM for melanoma cells) supplemented with 10% fetal bovine serum and incubated at 37°C, 5% CO_2_ for 20 h; two 24-well plates were prepared, one for the invasion assay and one for the survival assay. The next day, 2 × 10^6^ CFU of bacteria were added over the cells to reach a multiplicity of infection (MOI) of 10:1 and incubated at 37°C, 5% CO_2_ for 1 h. After three washes with PBS, a medium (RPMI for bladder cancer cells or DMEM for melanoma cells) containing 100 μg/mL gentamicin was added to each well and incubated for 1 h to kill extracellular bacteria. Then, each well was rewashed with PBS. For the invasion assay, tumor cells from one of the plates were immediately lysed with 0.5% Triton X-100 solution in PBS, followed by CFU determination by plating on LB-agar medium. For the survival assay, the other plate was incubated with medium (RPMI or DMEM) containing 20 μg/mL gentamicin and incubated for 4 h. Then, the tumor cells were washed with PBS and lysed for CFU determination. We performed three independent experiments, with three replicates from each strain.

### *In vitro* toxicity tests

The MTT assay evaluated the cytotoxic effect of the mutants as previously described [33]. A total of 1×10^4^ cells were seeded in 96-well plates with antibiotic-free medium (RPMI for bladder cancer cells or DMEM for melanoma cells) supplemented with 10% fetal bovine serum. Cells were incubated for 20 h at 37°C and 5% CO_2_. Then, the cells were washed with PBS and treated with 2 × 10^6^ CFU of bacteria to reach an MOI of 100:1. The plate was incubated at 37°C under 5% CO_2_ for 1 h. Cells were rewashed with PBS and incubated with medium (RPMI or DMEM) containing 100 μg/mL gentamicin for 2 h at 37°C under 5% CO_2_. Then, the cells were rewashed and incubated with a medium (RPMI or DMEM) containing 20 μg/mL of gentamicin for 24 h under appropriate culture conditions. After the incubation, the medium was removed, the cells were rewashed and the cells were re-incubated for 4 h with MTT (5 mg/mL) diluted in RPMI or DMEM medium. MTT diluted in the medium was discarded, and crystals were dissolved with dimethyl sulfoxide (DMSO-Sigma, Saint Louis, MO, United States). Absorbance was read at 570 nm. The viability of cells treated with the mutants was compared to that of untreated (control) cells. We performed three independent experiments, with three replications of each strain.

### Animals

6–8-week-old C57BL/6JUnib females were used in this study. All animals were obtained from the Multidisciplinary Center for Biological Research (CEMIB—UNICAMP). The animal care committee of Universidade Estadual de Campinas approved all experiments with mice under protocol numbers 5769-1/2021 and 5895-1/2021. The animals were kept under specific pathogen-free conditions. The mice were acclimatized for 2 weeks before the start of the experiments. Mice were 8 weeks old at the time of the first inoculations. The mice were shaved on the right flank 2 days before starting the experiments to facilitate subcutaneous inoculations.

### Treatment safety tests

The animals were randomly divided into four groups of five and injected subcutaneously with 60 µL in the right flank. Group I: mice received 10^5^ CFU of Δ*ihfABpmi* weekly for 2 weeks, two inoculations total. Group II: the animals received 10^6^ CFU of Δ*tolRA* weekly for 2 weeks. Group III: the animals received 10^7^ CFU of Δ*tolRA* weekly for 2 weeks. Group IV: mice received phosphate-buffered saline (PBS) weekly for 2 weeks. During the experiment, the animals were weighed thrice a week, and the appearance of any signs of disease (ocular discharge, piloerection, lethargy) was analyzed. They were euthanized 2 weeks after the last inoculation. In addition, parts of the liver, spleen, lung, and kidneys were collected and immediately fixed in 4% paraformaldehyde solution for organ damage analysis. Tissues were stained in H&E and analyzed under a Leica DM5500 B optical microscope (Wetzlar, Hesse, Germany). Two independent experiments were performed.

To quantify bacterial distribution and persistence at the end of the experiment, part of the liver, blood, and spleen were aseptically collected and homogenized in PBS with a tissue homogenizer (Omni Mixer Homogenizer, Vernon Hills, IL, United Stated), as previously described [29]. The homogenized tissues were plated on LB-agar, MacConkey, and SS with appropriate antibiotics to determine CFU. Two independent experiments were performed using 5 mice per group in each experiment.

### *In vivo* antitumor efficacy

The antitumor efficacy of the mutants was evaluated in a murine melanoma model. B16F10 cells were diluted in antibiotic-free and fetal bovine serum-free DMEM medium. B16F10 cells (3×10^6^) were inoculated subcutaneously into the dorsal flank region of C57BL/6JUnib mice. When the tumor reached 100 mm^3^ (10–12 days after tumor cell inoculation), the tumor-bearing animals were randomly assigned to groups (PBS, Δ*ihfABpmi,* and Δ*tolRA*), seven mice per group. Mice were inoculated intratumorally with 60 µL of mutant strains suspensions (10^5^ CFU of Δ*ihfABpmi* or 10^6^ CFU of Δ*tolRA*) or PBS once a week for 2 weeks. Tumor size was measured every 2–3 days, and tumor volume was calculated as previously described [19]. Two independent experiments were performed using 7 mice per group in each experiment.

Tumor-bearing mice that reached 2000 mm^3^ or showed signs of pain were euthanized to avoid suffering. The body weight of the mice was also measured every 2–3 days, and the mice were observed for any signs of disease throughout the treatment period. At the end of the experiments, the mice were euthanized by intraperitoneal injection of 5 mg/kg of xylazine and 40 mg/kg of ketamine, followed by cervical dislocation. Liver, spleen, kidney, and lung samples were collected, fixed in 4% formaldehyde, and stained with H&E for histopathological examination. The experiment was repeated twice.

To quantify bacterial distribution and persistence at the end of the experiment, tissues were weighed and homogenized with a tissue homogenizer (Omni Mixer Homogenizer, Vernon Hills, IL, United Stated), as previously described [29]. The mixed tissue suspension was plated in LB-agar, MacConkey, and SS with appropriate antibiotics to determine CFU. This experiment was performed twice.

The potential therapeutic antitumor of mutants was also evaluated intraperitoneally. Mice bearing B16F10 tumors (100 mm^3^) were treated intraperitoneally with 10^5^ CFU of Δ*ihfABpmi*, 10^6^ CFU of Δ*tolRA* or PBS once a week for 2 weeks. Tumor size was measured every 2–3 days. The mice were euthanized 19 days after starting the treatments. One independent experiment was performed using 5 mice per group.

### Immune response involved in the antitumor effect

To understand the immune response involved in the antitumor effect mediated by the Δ*ihfABpmi* mutant, mice bearing tumors of approximately 100 mm^3^ were inoculated intratumorally with 10^5^ CFU of Δ*ihfABpmi* or PBS. Four days later, the mice were euthanized. The tumor mass was divided into two parts, one for macrophage analysis by flow cytometry and another for analysis of the expression of genes involved in the antitumor response.

### Macrophage analysis by flow cytometry

A suspension of isolated cells was prepared from the tumor tissue. Tumor tissue was cut into small fragments and digested in 200 U/mL collagenase IV buffer at 37°C for 1 h, then passed through a 70 μm cell filter. The samples were incubated with fluorochrome-labeled antibodies [CD80-Pe, CD206-APC, CD11b-Percp, F4/80-Fitc (Elabscience. Houston, United States)] for 20 min at 4°C and 50,000 events were analyzed using the cytometer of NovoCyte flow using the following panel macrophage (F4/80+ CD11b+), M1 macrophage (F4/80+ CD80^+^) and M2 macrophage (F4/80+ CD260+). The data were analyzed with the software NovoExpress 1.5.0 software.

### Quantitative RT-PCR (qRT-PCR) for the detection of gene expression

mRNA expression was analyzed by qRT-PCR. Total RNA extraction from tumor tissue was performed using a Direct-zol RNA MiniPrep Plus kit (Zymo Research, Irvine, CA, United States) and Trizol reagent (Invitrogen, United States) according to the manufacturer’s instructions. RNA integrity was confirmed by agarose gel followed by ethidium bromide staining. RNA purity and concentration were verified using NanoDrop 2000c (Thermo Scientific, United States). The RNA was treated with DNase I Amplification grade (Sigma-Aldrich. Louis, MO, United States) to eliminate genomic DNA contamination. As a contamination control, a PCR was performed on all samples to verify the absence of genomic DNA. Reverse transcription was performed to synthesize cDNA using the High-Capacity cDNA Reverse Transcription kit (Applied Biosystems. United States). Then, the real-time PCR reaction was performed using the 2x qPCRBioSyGreen Mix Separate-Rox kit (PCRBIOSYSTEMS. Wayne, Pennsylvania, United States), according to the manufacturer’s instructions, in a MicroAmp Optical 96-Well Reaction Plate microplate (Thermo Scientific, United States), using the StepOnePlus Real-Time PCR System (Applied Biosystems, United States). The dissociation Curve was made to verify the specificity and quality of the primers used. The mRNA expression was normalized to the expression of β-actin and GAPDH, which were used as endogenous controls. Relative mRNA expression levels were calculated using the 2^−ΔΔCT^ method. The sequences of the primers used are shown in Supplementary Table S2.

### Statistical analysis

Statistical analysis was performed using GraphPad Prism version 8 (GraphPad, San Diego, CA, United States). One-way ANOVA followed by Dunnett’s test was used in experiments with three or more experimental groups. Student’s t-test was used to analyze data from experiments from two experimental groups. Data were represented as the mean ± Standard Deviation of the mean (SEM), and *p* < 0.05 was considered statistically significant.

## Results

### *In vitro* growth of mutants and attenuation of virulence in the *Galleria mellonella* model

**FIGURE 1:**
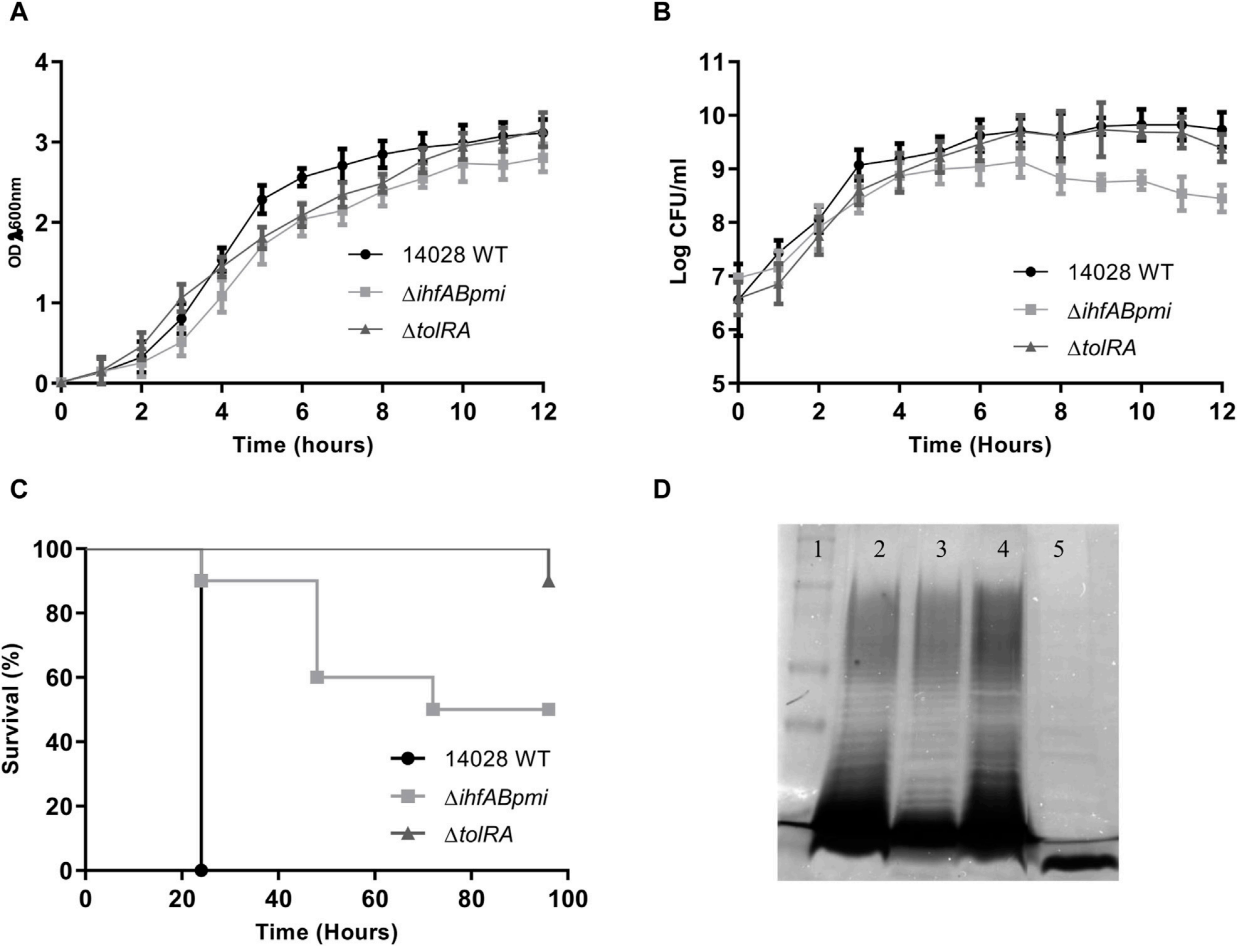
Bacterial growth and virulence of mutants. **(A)** Growth of the mutants and wild-type strain, monitored for 12 h by measuring the OD (λ 600 nm); **(B)** number of colony forming units (CFU) per ml of the mutants and wild-type strain monitored every hour. Growth curves were performed three times. The graph shows the mean ± SD of three independent experiments. **(C)** Virulence of mutants and wild-type strains in the *Galleria mellonella* model. Larvae were inoculated with 10^4^ CFU in the last proleg, and survival was observed for 96 h. **(D)** Electrophoretic profile of lipopolysaccharides (LPS). Polyacrylamide gel stained with silver to confirm bacterial LPS integrity. 1. Dual color molecular weight standard (Bio-rad); 2. *Salmonella enterica* Typhimurium 14028; 3. *S. enterica* Typhimurium 14028 Δ*ihfABpmi*; 4. *S. enterica* Typhimurium 14028 Δ*tolRA*. 5. *Pseudomonas aeruginosa* (Δ*wzz*). The graphs show representative values from three independent experiments.

First, we verified the potential effects of gene deletions on bacterial growth by comparing the mutants (Δ*ihfABpmi* and Δ*tolRA*) with the parental strain 14028 WT. For *in vitro* growth in LB broth (37°C, 150 rpm), we observed similar OD_600_ values between the mutants and the parental strain (Figure 1A). However, analysis of colony forming units (CFU) number counts showed that the growth rate of Δ*ihfABpmi* was lower than that of 14028 WT (Figure 1B), and Δ*ihfABpmi* had a lower number of CFUs in the stationary phase of growth as compared to 14028 WT. Δ*tolRA* showed a growth pattern similar to 14028 WT (Figure 1B).

Next, we investigated whether the deleted genes affected the virulence of 14028 WT by inoculating *G. mellonella* larvae with 1 × 10^6^ CFU of Δ*ihfABpmi*, Δ*tolRA*, and 14028 WT (Figure 1C and Supplementary Figure S1). We observed lower mortality of larvae inoculated with the mutants than with the parental strain. Here, 90% and 50% of larvae inoculated with Δt*olRA* and Δ*ihfABpmi*, respectively, survived 96 h post-inoculation, while all larvae inoculated with 14028 WT died within 24 h of inoculation. These results suggest that the Δ*ihfABpmi* and Δ*tolRA* mutants are virulence-attenuated.

Electroporation, used for constructing mutants, can result in the selection of mutant colonies with incomplete LPS. However, LPS is critical for bacterial immunogenicity. Therefore, we investigated the LPS integrity of the mutants by performing LPS preparations on polyacrylamide gels, followed by periodic acid oxidation and silver staining. We observed similar LPS profiles of the mutants and the wild-type strain, suggesting that the constructed mutants had a complete LPS layer (Figure 1D).

### Invasion and survival capability of mutants in cancer cells

**FIGURE 2:**
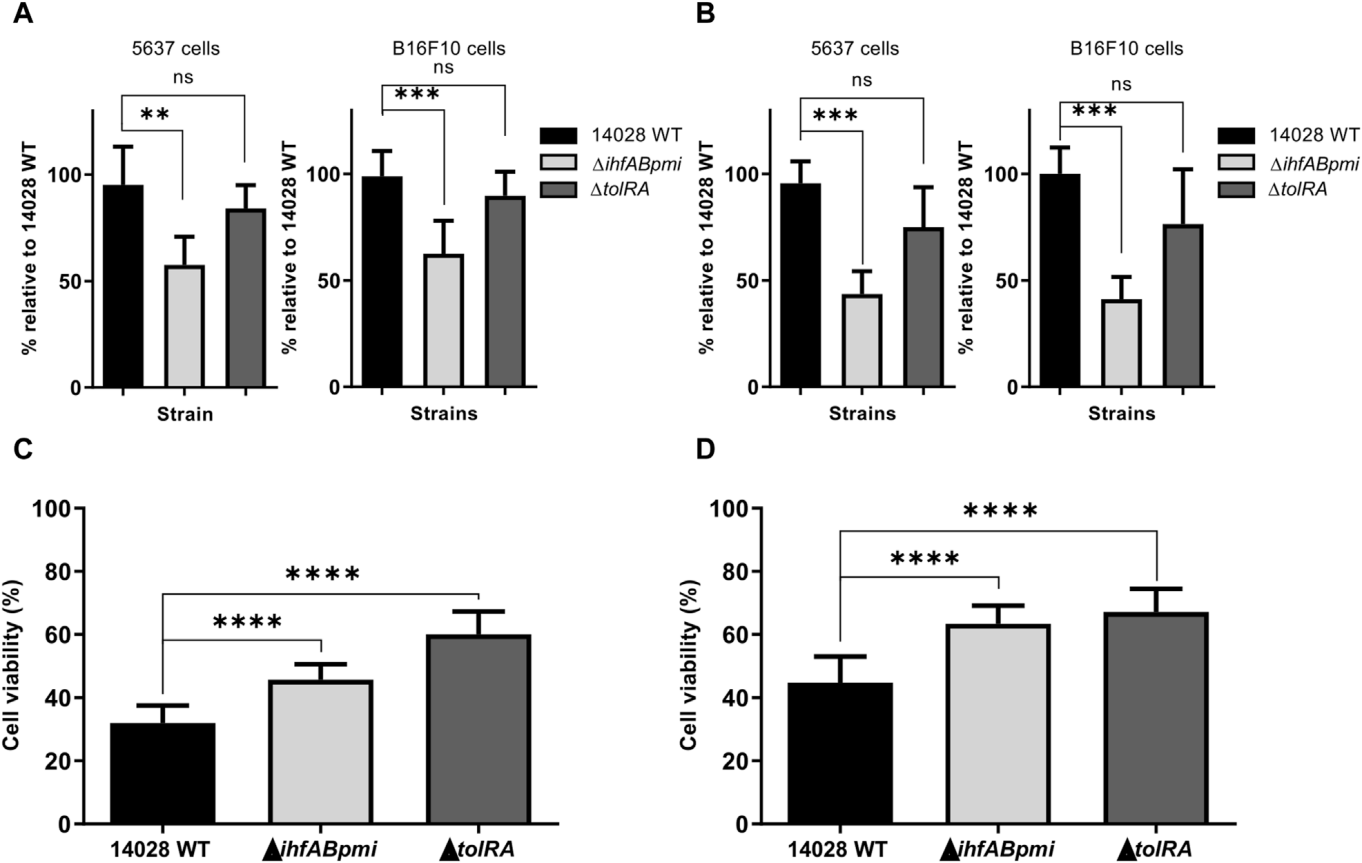
Invasion, survival, and cytotoxicity of mutant strains in bladder carcinoma (5,637) and melanoma (B16F10) cells. Cancer cells were infected *in vitro* with mutant strains for 2 and 6 h at an MOI of 10:1, after which the ability of invasion **(A)** and survival **(B)** of the mutant strains in cancer cells was determined by measuring intracellular CFU. 14028 WT was used as a positive control. Percentage of viable 5,637 **(C)** and B16F10 **(D)** cancer cells after 24 h of bacterial strain infections. The results were normalized to those of the uninfected cells. Results are represented as mean ± SD of three independent experiments performed in triplicate wells. Statistical significance was determined by one-way ANOVA followed by Multiplex Comparison Test *p < 0.05; **p < 0.01; ***p < 0.005; ****p < 0.0001.

We determined whether gene deletion affected the invasiveness and survival of 14028 WT in tumor cells by infecting two cancer cell lines with the mutant and 14028 WT strains for 2 and 6 h, followed by determining the number of intracellular CFU after tumor cell lysis. In B16F10 murine melanoma cells, Δ*tolRA* showed a similar invasion and survival profile to the parental strain. However, the Δ*ihfABpmi* mutant showed a statistically significant reduction in invasiveness and survival, and similar results were observed with bladder carcinoma 5,637 cells (Figures 2A, B).

### Cytotoxicity of *S. enterica* Typhimurium mutants in tumor cells

In addition to invasion and survival, potential *S. enterica* Typhimurium mutants must also exert a cytotoxic effect. We assessed the *in vitro* antitumor potential of the mutants by performing the colorimetric assay with 3-(4,5-dimethylthiazol-2-yl)-2,5-diphenyltetrazolium bromide (MTT) in B16F10 melanoma and 5,637 bladder carcinoma cells (Figures 2C, D). The MTT assay was performed with previously proposed modifications for the elimination of bacterial contribution to MTT reduction [33]. Cancer cells without the addition of bacteria were used as a negative control (100% viability), while cells treated with 14028 WT were used as a positive control. We observed that 14028 WT, Δ*tolRA* mutant and Δ*ihfABpmi* mutant significantly decreased the viability of both cell lines (melanoma and bladder carcinoma cells). Treatment with 14028 WT decreased viability by 55%–68%, Δ*ihfABpmi* mutant treatment decreased viability by 36%–54%, while the Δ*tolRA* mutant decreased viability by 32%–40%. B16F10 melanoma cells were more resistant to the cytotoxic effect of both mutants and the parental strain, likely due to their aggressive and metastatic phenotype.

### Safety analysis of treatment with attenuated *S. enterica* Typhimurium mutants

**FIGURE 3:**
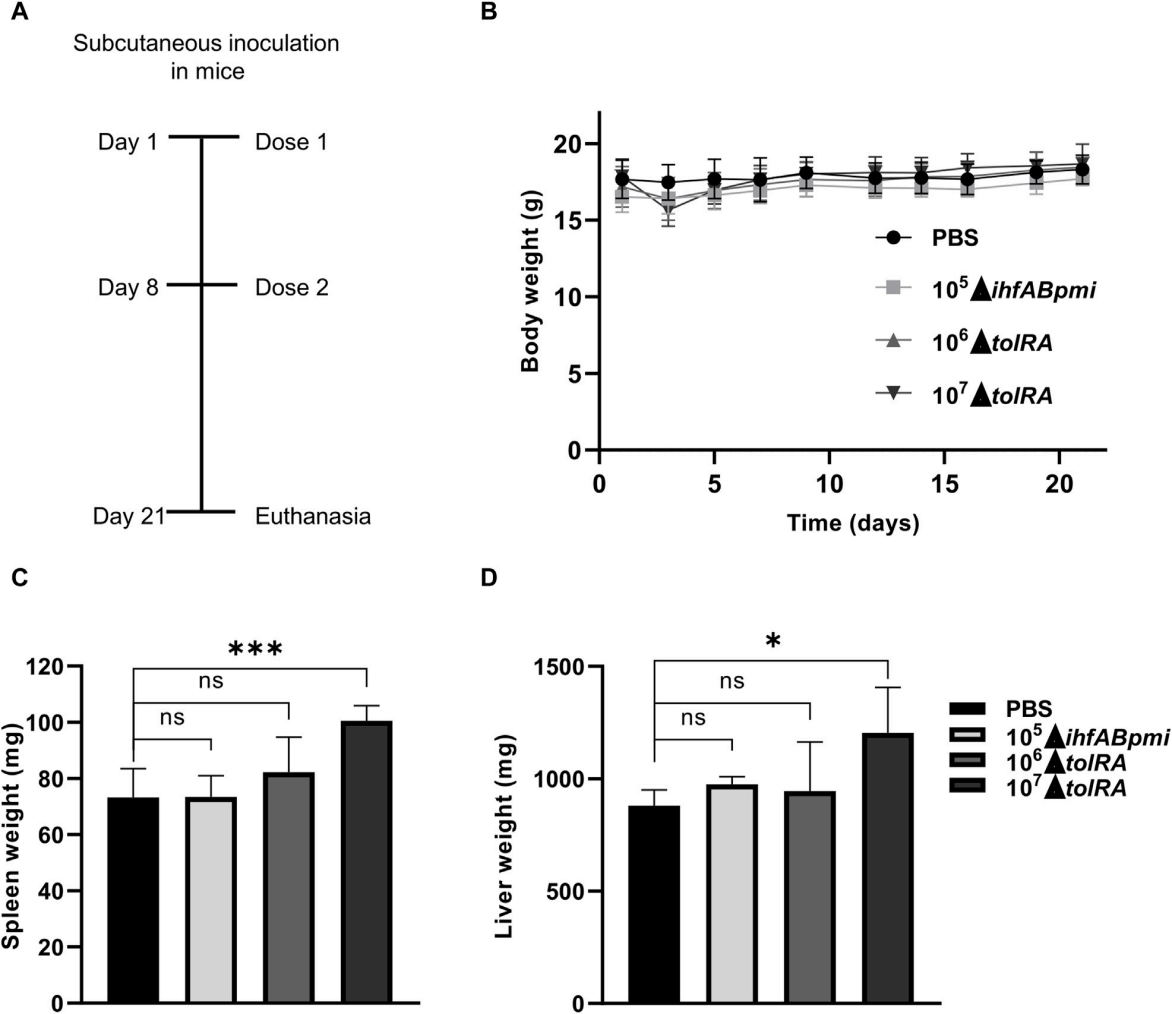
Safety assessment of treatment with attenuated mutants of *Salmonella enterica* Typhimurium. **(A)** Experimental design to test the toxicity and safety of mutants in mice inoculated by the subcutaneous route. **(B)** Body weight of mice inoculated with attenuated mutants throughout the experiment. Weight of spleens **(C)** and livers **(D)** of mice inoculated with attenuated mutants. In the graphics, the error bars represent the average ± SD of 5 mice per group. Data are from one experiment representative of two independent experiments. Each experiment performed with 5 mice per group. Statistical significance was determined by one-way ANOVA followed by Multiplex Comparison Test *p < 0.05; **p < 0.01; ***p < 0.005; ****p < 0.0001.

We assessed treatment tolerance with attenuated mutants of *S. enterica* Typhimurium by inoculating healthy mice subcutaneously with the mutants once a week for 2 weeks, for a total of two doses (the experimental design is shown in Figure 3A). The mutant Δ*ihfABpmi* dose (10^5^ CFU) was chosen based on our previous studies that showed that the double mutant Δ*ihfAB* is an attenuated strain and 10^5^ CFU of the Δ*ihfAB* mutant is a safe and efficient dose to treat bladder cancer in mice (unpublished data). Based on these results, we chose 10^5^ CFU of the triple mutant Δ*ihfABpmi* as the experimental dose here. A previous study reported that the Δ*tolRA* mutant is highly attenuated [28], and our results in *G. mellonella* further confirmed this attenuation (Figure 1C and Supplementary Figure S1). Thus, we tested two doses of 10^6^ and 10^7^ CFU of the Δ*tolRA* mutant in safety analysis experiments.

The body weight of the mice was determined as a sign of general health. After the first inoculation, we observed that mice inoculated with 10^7^ CFU of the Δ*tolRA* mutant lost ±2 g (12% of body weight) with signs of disease, such as eye discharge, piloerection, and lethargy. However, 1 week after the first inoculation with 10^7^ CFU of the Δ*tolRA* mutant, the mice regained weight, and the disease symptoms disappeared (Figure 3B). In mice inoculated with PBS, 10^6^ CFU of the Δ*tolRA* mutant, or 10^5^ CFU of the *ΔihfABpmi* mutant, we did not observe weight loss or signs of systemic disease such as ocular discharge, piloerection, lethargy throughout the experiment (Figure 3B). Despite the symptoms, no mortality was observed in the mice throughout the experiment (Table 1).

**TABLE 1:** Safety trial of treatment with subcutaneous attenuated mutants in C57BL/6JUnib mice (5 per group).

Groups	CFU	Survivors/inoculates
PBS		5/5
*ΔihfABpmi*	10^5^	5/5
*ΔtolRA*	10^7^	5/5
*ΔtolRA*	10^6^	5/5

Data are from one experiment representative of two independent experiments. Each experiment performed with 5 mice per group. In both experiments, survival was 100% in all groups analyzed.

The mice were euthanized 21 days after the initial inoculation, and the organs were collected for bacterial persistence and histopathological analysis. To analyze bacterial distribution and persistence at the end of the experiment, homogenized or macerated blood samples from the liver and spleen were diluted and plated on LB agar, SS, and MacConkey with appropriate antibiotics for subsequent CFU counting of our mutants. However, no bacterial colonies were detected in any of the three media tested or any tissue group, even when samples were plated without dilution. The fact that no mutants were isolated suggests that the bacteria cannot persist in these organs 21 days after the first inoculation and 15 days after the second inoculation.

Macroscopic examination of the organs showed hepatomegaly and splenomegaly in the group inoculated with 10^7^ CFU of the Δ*tolRA* mutant. Significant differences in spleen and liver weights were observed in mice inoculated with 10^7^ CFU of the Δ*tolRA* mutant, as compared to the group inoculated with PBS (Figures 3C, D). However, we did not observe significant differences between organ weights of mice inoculated with 10^6^ CFU of the Δ*tolRA* mutant or 10^5^ CFU of the Δ*ihfABpmi* mutant compared with mice inoculated with PBS (Figures 3C, D).

Microscopic analysis of spleen, liver, lung, and kidney samples from mice inoculated with PBS revealed typical histological structures (Supplementary Figure S2). The spleen, liver, kidney, and lung of mice inoculated with the Δ*ihfABpmi* mutant showed no signs of tissue damage. However, histological changes related to mild inflammation were observed in the liver, lung, and spleen. Interestingly, we also observed an increase in the megakaryocyte number in the spleen of mice inoculated with the Δ*ihfABpmi* mutant as compared to that in mice inoculated with PBS. Megakaryocytes have previously been related to inflammation [34]. Despite these effects, no histological architectural changes were observed in the evaluated organs, suggesting that subcutaneous inoculation of 10^5^ CFU of the Δi*hfABpmi* mutant is safe for use as a treatment.

### Antitumor efficacy of attenuated *S. enterica* Typhimurium mutants in the murine melanoma model

**FIGURE 4:**
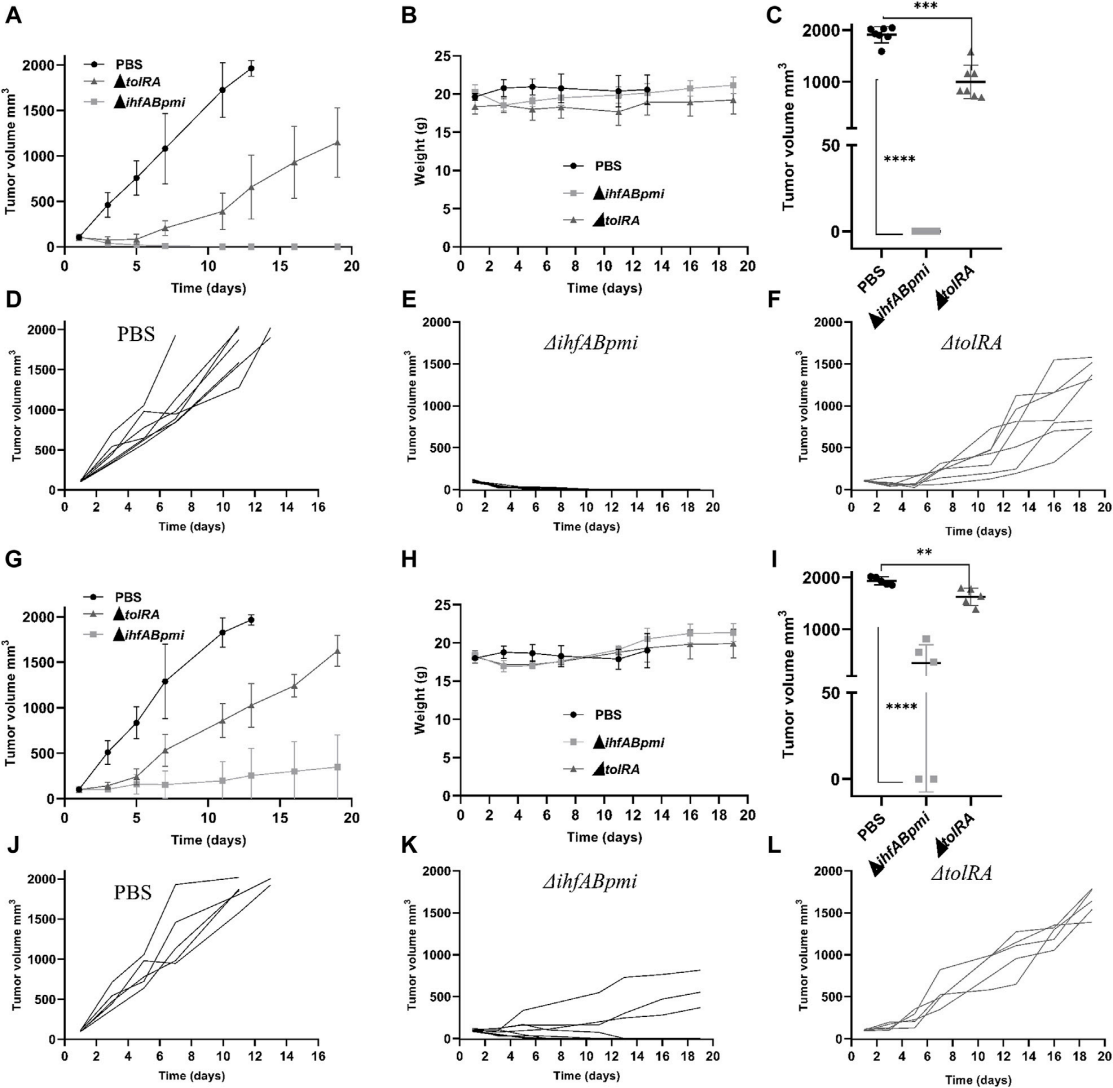
Antitumor efficacy of attenuated mutants of *Salmonella enterica* Typhimurium in a B16F10 subcutaneous tumor model. C57BL/6JUnib mice were inoculated with B16F10 cells (3 × 10^6^), subcutaneously in the dorsal flank region. When the tumor reached 100 mm^3^ (10–12 days after tumor cell inoculation), 10^6^ CFU of the Δ*tolRA* mutant, 10^5^ CFU of the Δ*ihfABpmi* mutant or PBS was injected intratumorally (*n* = 7) or intraperitoneal (*n* = 5) once a week for 2 weeks (*n* = 7). **(A)** Tumor growth after starting treatments intratumorally. **(B)** Weight of mice during treatments intratumorally. Data reported in graphs A and B are average values ± SD. **(C)** Tumor size at the endpoint of the treatments intratumorally. **(D–F)** Kinetics of individual tumor growth in groups treated intratumorally with PBS, Δ*ihfABpmi* mutant, and Δ*tolRA* mutant, respectively. Day one is considered when the tumor reaches 100 mm^3^. Two independent experiments were performed, using seven mice per group. **(G)** Tumor growth after starting treatments intraperitoneal. **(H)** Weight of mice during treatments intraperitoneal. Data reported in graphs **(G, H)** are average values ± SD. **(I)** Tumor size at the endpoint of the treatments intraperitoneal. **(J–L)** Kinetics of individual tumor growth in groups treated intraperitoneal with PBS, Δ*ihfABpmi* mutant, and Δ*tolRA* mutant, respectively. Day one is considered when the tumor reaches 100 mm^3^. One experiment was performed, using five mice per group. Statistical significance was determined by one-way ANOVA followed by Multiplex Comparison Test *p < 0.05; **p < 0.01; ***p < 0.005; ****p < 0.0001.

Based on the safety analysis described above, we performed the antitumor efficacy tests with 10^6^ CFU of the Δ*tolRA* mutant and 10^5^ CFU of the Δ*ihfABpmi* mutant since, at these concentrations, no weight loss, splenomegaly, or hepatomegaly were observed, suggesting that these mutants are well-tolerated in mice at these concentrations. The antitumor efficacy of the mutants was evaluated in terms of the survival rate of the mice and the ability to reduce tumor mass. B16F10 cells (3 × 10^6^) were implanted subcutaneously in the dorsal flank region of female C57BL/6JUnib mice. When the tumor reached 100 mm^3^ (10–12 days after tumor cell inoculation), mice bearing B16F10 tumors were treated intratumorally or intraperitoneal with 10^6^ CFU of the Δ*tolRA* mutant or 10^5^ CFU of the Δ*ihfABpmi* mutant. Mice bearing B16F10 tumors inoculated with PBS were used as a negative control. As shown in Figure 4, both the Δ*tolRA* mutant and the Δ*ihfABpmi* mutant treatments reduced tumor growth compared with the PBS-treated group (control), and treatment with the Δ*ihfABpmi* mutant was more efficient in reducing melanoma tumors than treatment with the Δ*tolRA* mutant.

Figures 4C, I show the tumor size at the end point of the experiment (19 days after the first dose of treatment with the mutants). Intratumoral treatment with the Δ*ihfABpmi* mutant eliminated the tumor from all mice, leaving only a scar at the tumor site (Figure 4C). Moreover, we also observed that the Δ*ihfABpmi* mutant completely reduced the tumor mass of all mice 6 days after the first dose of treatment, which did not grow back in the following 15 days (Figure 4E). Intraperitoneal treatment with the Δ*ihfABpmi* mutant eliminated the tumor in two of the five treated mice (Figure 4I). On the other hand, although intratumor or intraperitoneal treatment with the Δ*tolRA* mutant inhibited tumor growth compared to the PBS control, it did not eliminate tumors in mice.

All mice inoculated with the mutants survived until the endpoint of the experiment. However, the mice in the PBS group were euthanized 1 week before the end point of the experiment to avoid further suffering due to substantial tumor growth. We did not observe any significant differences between the weight of the mice inoculated with the mutants and the PBS group (Figures 4B, H). We also did not observe any signs of disease in mice intraperitoneally or intratumorally treated with the Δ*ihfABpmi* mutant.

In the endpoint of the experiment intratumorally, blood, liver, spleen, and tumor samples were homogenized or macerated and plated on LB agar, SS, and MacConkey for subsequent CFU counting. However, no bacterial colonies were detected in the analyzed groups (mice treated with PBS, Δ*ihfABpmi*, or Δ*tolRA*), even when samples were plated without dilution. The non-isolation of the mutants suggests that the bacteria either cannot persist in these organs or get eliminated by the immune system of the mice. It would be interesting to determine the colonization efficiency of the two mutants tested in the tumor microenvironment to see if differences in antitumor activity can be attributed to bacterial fitness. Future analyses will include the analysis of bacterial colonization after 24, 48 and 72 h of treatment with the mutants.

**FIGURE 5:**
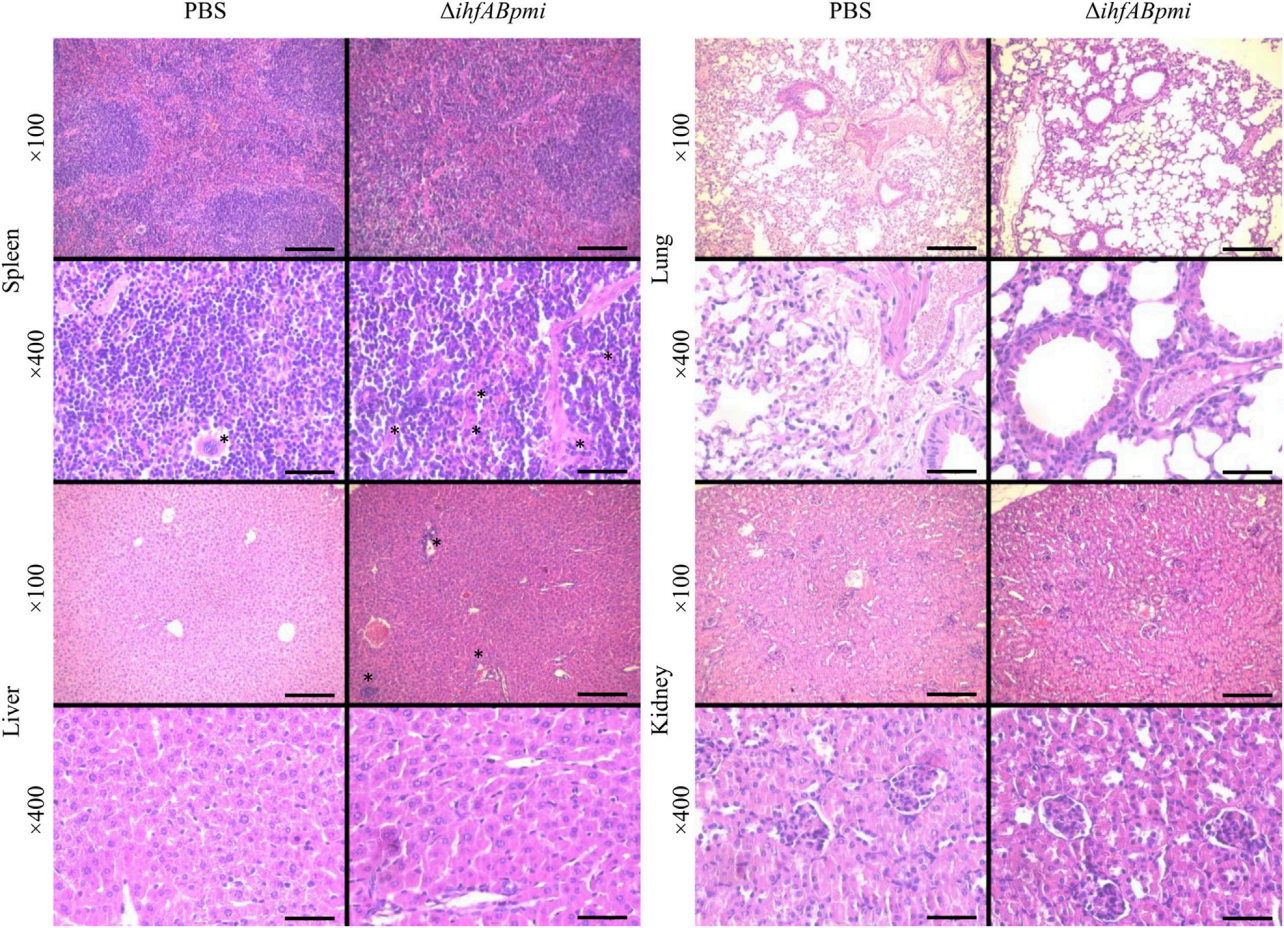
Histological analysis of normal tissues. Tumor-bearing mice were treated with 10^5^ CFU of Δ*ihfABpmi* mutant or PBS, twice a week for 2 weeks. After 1 week of the last inoculation, the spleen, liver, lung, and kidney were collected for histological analysis using H&E staining of organ sections. Slight inflammation was observed in the liver and spleen of mice treated with Δ*ihfABpmi* mutant. The asterisks indicate the megakaryocytes in the spleens and inflammatory foci in the liver. Scale bar: 200 μm for ×100 and 50 μm for ×400.

Hematoxylin and Eosin (H&E) staining of tissues revealed inflammatory foci (infiltration of mononuclear and polymorphonuclear cells) in the liver and spleen of mice treated with the Δ*ihfABpmi* mutant (Figure 5). However, we did not observe pathological changes in the architecture of the analyzed organs.

### Analysis of the immune response involved in the antitumor effect

The immunological responses underlying the antitumor effects of *S. enterica* Typhimurium remain poorly understood. Recent evidence suggests the involvement of cells of the innate immune system, such as macrophages, and the production of pro-inflammatory cytokines in tumor elimination [15]. We used the Δ*ihfABpmi* mutant to analyze the immune responses involved in its antitumor response because treatment with the Δ*ihfABpmi* mutant showed a more significant tumor inhibition than treatment with the Δ*tolRA* mutant. Mice were euthanized, and tumor tissue was collected 4 days after treatment with 10^5^ CFU of the Δ*ihfABpmi* mutant, as the Δ*ihfABpmi* mutant rapidly inhibited tumor growth.

**FIGURE 6:**
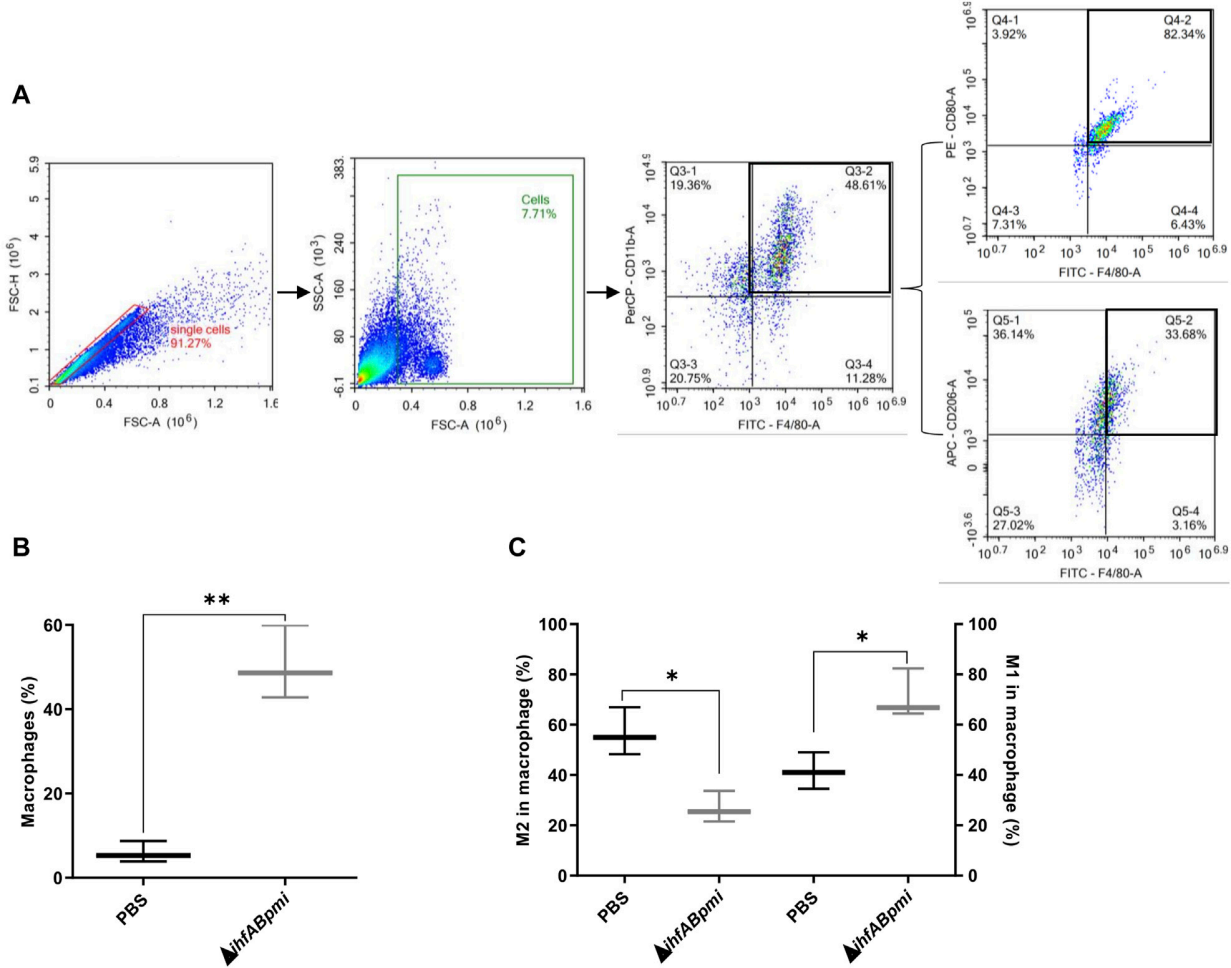
Immunophenotyping of macrophages infiltrated in B16F10 tumors. **(A)** Representative gating strategy to identify macrophage (F4/80+ CD11b+), M1 macrophage (F4/80+ CD80^+^) and M2 macrophage (F4/80+ CD206+). **(B)** Macrophage population and **(C)** Macrophage phenotype (M1 or M2 type) after intratumoral treatment with 10^5^ CFU of the Δi*hfABpmi* mutant or PBS in a murine model of melanoma (three mice per group). Results are presented as the mean ± SD (*n* = 6). Statistical significance was calculated by Student’s t-test. *p < 0.05; **p < 0.01.

Tumor cell suspensions prepared from tumor tissue were labeled and analyzed using the following panel macrophage (F4/80+ CD11b+), M1 macrophage (F4/80+ CD80^+^) and M2 macrophage (F4/80+ CD260+). Flow cytometric analysis showed significant difference between the proportion of macrophages from mice treated with the Δ*ihfABpmi* mutant and mice treated with PBS (Figure 6B). Next, we examined the phenotype of intratumoral macrophages, and observed that treatment with the Δ*ihfABpmi* mutant significantly reduced the proportion of M2-type macrophages and increased the proportion of M1-type macrophages compared to PBS-treated mice (Figure 6C). This suggests that the tumor elimination ability of Δ*ihfABpmi* is associated with the induction of the accumulation of macrophages with antitumor phenotype and the reduction of pro-tumor immunosuppressive macrophages.

**FIGURE 7:**
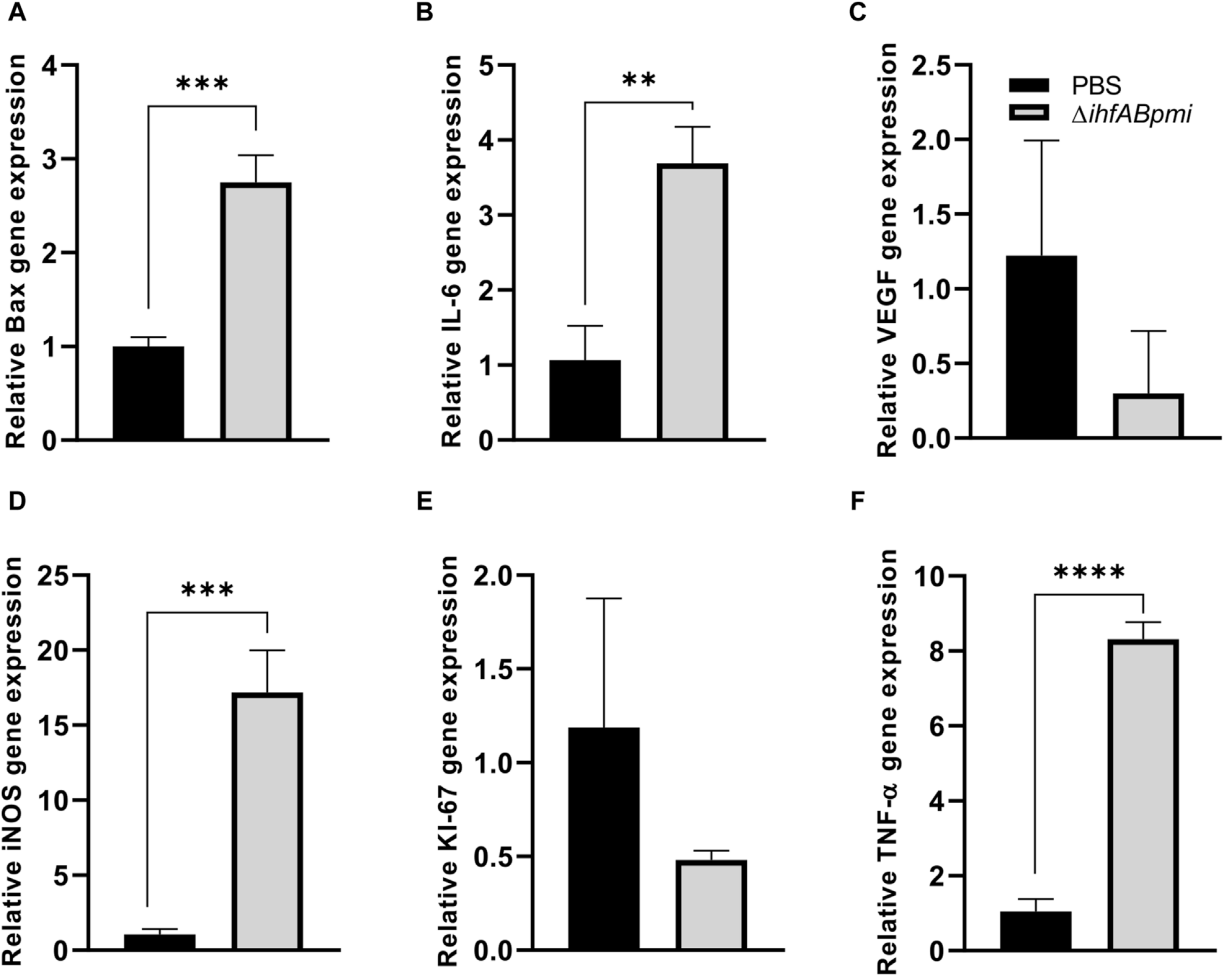
Effect of the Δ*ihfABpmi* mutant on gene expression. The relative level of RNA expression of Bax **(A)**, IL-6 **(B)**, VEGF **(C)**, iNOS **(D)**, Ki-67 **(E)**, and TNF-α **(F)** genes. Tumor tissue from B16F10 tumor-bearing mice was collected after 4 days of an intratumoral injection with 10^5^ CFU of the Δ*ihfABpmi* mutant or PBS (*n* = 6). Results are presented as the mean ± SD. Statistical significance was calculated by Student’s t-test. *p < 0.05; **p < 0.01; ***p < 0.005; ****p < 0.0001.

We evaluated the expression of cell proliferation genes (Ki-67), angiogenesis (VEGF), apoptosis (Bax), and pro-inflammatory (IL-6, TNF-α, and iNOS) markers to further investigate the immune responses underlying the antitumor effects of Δ*ihfABpmi*. Tumor tissue from mice treated with the *ΔihfABpmi* mutant or PBS was collected after 4 days to measure the levels of mRNA expression using qRT-PCR. We observed that treatment with the Δ*ihfABpmi* mutant induced a statistically significant upregulation of the mRNA levels of Bax, IL-6, TNF-α, and iNOS (Figures 7A, B, D, F, respectively). Thus, pro-inflammatory cytokines secreted by immune cells such as macrophages can contribute to tumor cell death and trigger a strong tumor-specific immune response [35].

## Discussion

Bacteria-based anticancer therapy is a promising option for treating cancer [22, 36]. Facultative anaerobic bacteria such as *S. enterica* are the focus of anticancer research due to their natural ability to attack tumors with variable oxygen concentrations and their immunogenicity, which leads to the activation of the immune system to destroy tumors in animal models [37, 38]. However, *S. enterica* is also a pathogen that causes Salmonellosis in humans [39]. Therefore, their virulence must be attenuated for *S. enterica* strains to be considered in cancer therapy to ensure safe use [22].

The virulence of *S. enterica* can be attenuated by mutating or eliminating pathogenicity genes or genes essential for survival [19, 40, 41]. However, the mutation of these genes can also compromise their anticancer activity and their ability to invade and destroy tumor cells [42]. For example, deletion of the genes for LPS biosynthesis (Δ*rfG* and Δ*rfD*) decreases the intrinsic antitumor effect of *S. enterica* Typhimurium 14028 (Frahm et al., 2015). Strains KST0651 (∆*relA* ∆*spoT*) and KST0649 (∆*ptsI* ∆*crr*) lost the ability to replicate in macrophages and epithelial cells [43, 44]. The low yield of the VNP20009 strain, modified in lipid A (Δ*msbB*Δ*purI*), as evidenced by the lack of tumor colonization and antitumor activity in clinical trials, has been attributed to the deletion of the *msbB* gene [23, 24, 45]. Therefore, careful selection of *S. enterica* strains is required to maintain their tumor-attacking ability while achieving their attenuation.

We demonstrated the antitumor efficacy of two strains of *S. enterica* Typhimurium, whose antitumor potential has not been explored yet. The strains were designed by deleting chromosomal genes. The double mutant Δ*tolRA* lacks two proteins critical for maintaining the integrity of the bacterial membrane (TolR and TolA), and the triple mutant Δ*ihfABpmi* lacks two proteins, IHF, a nucleoid-associated protein that also functions as a transcriptional regulator and is involved in the expression of pathogenicity genes, and 6-phosphomannose isomerase, a key enzyme for lipopolysaccharide O production. Of these mutants, the Δ*tolRA* mutant was highly attenuated, but its ability to eliminate melanoma tumors was compromised, at least in the mouse model. The Δ*ihfABpmi* mutant, in turn, showed less attenuation than the Δ*tolRA* mutant, but maintained its tumor-eliminating ability.

The TolR and TolA proteins are part of the Tol-Pal system, a critical multiprotein complex for maintaining the integrity of Gram-negative bacteria’s cell envelope associated with bacterial virulence [46, 47]. The Tol-Pal system crosses the inner, periplasm, and outer membrane [48]. TolR and TolA are anchored to the inner membrane through a single region near the N-terminus [49]. Inactivation of any of the Tol-Pal system genes negatively affects outer membrane integrity, results in leakage from the periplasm, increases susceptibility to toxic compounds, and increases outer membrane vesicle production [46].

In addition to maintaining the cell envelope structure, TolR and TolA proteins also have other biological functions. In *E. coli*, the TolR protein is involved in the retrograde transport of phospholipids. In *Shigella flexneri*, strains lacking the *tolR* gene are more sensitive to antibiotics and bile salts and are less virulent [50, 51]. In *S. enterica* Typhimurium, the TolR protein has motility-related functions, and deletion of the *tolR* gene significantly increases outer membrane vesicle production [52, 53].

Deletion of the gene encoding the TolA protein in *S. enterica* Typhimurium increases its sensitivity to bile salts and reduces motility and bacterial load in the spleen, liver, Peyer’s patches, and lymph nodes compared with *S. enterica* wild-type. *Salmonella enterica* Typhimurium Δ*tolA* is considered highly attenuated in mice and *G. mellonella* and is more sensitive to the complement system than wild-type strains, a feature that has also been reported for mutants lacking *tolR* [50, 52, 54, 55]. Our virulence results for the Δ*tolRA* mutant are consistent with previous reports. In the *G. mellonella* model, the Δ*tolRA* mutant was highly attenuated as 90% of larvae inoculated with the Δ*tolRA* mutant survived. In contrast, all larvae inoculated with the wild-type strain died 24 h after inoculation (Figure 1C).

The attenuation and hypervesiculation profiles of mutants lacking the *tolR* and *tolA* genes made these strains and the outer membrane vesicles isolated from these mutants a research focus in the development of vaccines against *S. enterica*, *Mycobacterium tuberculosis*, *E. coli,* and SARS-CoV-2 [28, 54, 56, 57]. However, its anticancer potential has not been investigated yet. In this study, we investigated whether *S. enterica* Typhimurium Δ*tolRA* double mutant possesses antitumor activity *in vitro* and *in vivo*. The Δ*tolRA* mutant exhibits essential features for a potential antitumor strain, such as strong attenuation of virulence (Figure 1C), ability to invade and survive similar to the wild-type strain (Figures 2A, B), and toxicity in bladder cancer and melanoma cells (Figures 2C, D). The main feature of the Δ*tolRA* mutant observed in this study was its ability to inhibit melanoma growth in a murine model. The Δ*tolRA* mutant was not sufficient to eradicate the tumor mass (Figure 4). The high attenuation of this strain likely compromised its antitumor efficacy.

To date, none of the analyzed strains is capable of eradicating human tumors, so research into new antitumor strains is necessary to obtain more effective and safer strains that can be used in anticancer treatment [23, 24, 45]. We previously showed that the mutant for the *ihfA* gene of *S. enterica* Typhimurium attenuates virulence and reduces tumor mass in a murine model. However, this strain exhibited high toxicity *in vivo*, with 70% of mice treated intratumorally with *S. enterica* Typhimurium Δ*ihfA* dying 15 days after inoculation with a 10^5^ CFU dose [19].

The *ihfA* and *ihfB* genes encode the α and β subunits of the heterodimeric IHF protein [58, 59]. IHF binds to specific transcriptional promoters and induces their bending >120°. Influencing transcription by facilitating the interaction between RNA polymerase and regulatory proteins [60, 61]. A recent study in *Pseudomonas putida* revealed that IHF influences homologous recombination and mutagenic processes and that recombination and mutation rates are lower in mutants for the IHF protein [62]. In *S. enterica* Typhimurium, IHF positively controls the expression of several virulence genes and cell invasion [63]. Strains lacking *ihfA* and *ihfB* are attenuated, leading to global transcriptional dysregulation [64, 65]. Previous studies have shown that the homodimeric forms of IHF (αα or ββ) are biologically active but less stable [66]. However, mutations lacking *ihfA* or *ihfB* are still capable of producing homodimeric proteins which might explain the increased toxicity of the single Δ*ihfA* mutant in our previous study [19].

To solve the toxicity problems of the Δ*ihfA* mutant, we constructed an IHF-deficient strain (homodimer or heterodimer) by deleting *ihfA* and *ihfB*. The double mutant *ihfA* and *ihfB* of *S. enterica* Typhimurium 14028 did not presented toxicity as exhibited by the Δ*ihfA* single mutant [19]. We also showed that the double mutant *ihfA* and *ihfB* of *S. enterica* Typhimurium 14028 and violacein exerted an antitumor effect on bladder cancer in a murine model (unpublished data). Considering these results and to improve the safety and attenuation of the double mutant of *S. enterica* Typhimurium 14028 Δ*ihfA*Δ*ihfB*, we constructed a new triple mutant in this study by additionally deleting the *pmi* gene.

The *pmi* gene encodes 6-phosphomannose isomerase, an enzyme that performs the reversible interconversion of fructose-6-phosphate to mannose-6-phosphate, a precursor of mannose GDP. Since GDP-mannose is required for the synthesis of the O antigen side chain of LPS, strains with a *pmi* gene mutation grow in the absence of mannose but cannot synthesize the O antigen. These mutants can only produce complete LPS in the presence of exogenous mannose [67, 68]. Mutants with a deletion in the *pmi* gene have already been studied previously, as they allow obtaining strains with regulated delayed attenuation, a strategy implemented for constructing safe and immunogenic vaccine strains [68–70]. Δ*pmi* mutants are grown in a mannose-supplemented culture medium, allowing optimal host colonization in the initial stages of invasion. After several generations of *in vivo* growth, the O antigen is gradually lost, and the Δ*pmi* mutant is attenuated by the absence of mannose in animal tissues [68].

In this study, we deleted the *pmi* gene based on previous studies demonstrating that these mutants are attenuated and immunogenic, with the advantage of ceasing the expression of complete LPS *in vivo* [67]. This characteristic gives the strain greater security for the host since strains with incomplete LPS are more susceptible to the immune system [67, 70].

Our results suggest that the Δ*ihfABpmi* mutant is an attenuated strain with antitumor potential that balances attenuation and antitumor efficacy. Although the triple mutant Δ*ihfABpmi* is an attenuated strain, its attenuation profile was lower than the attenuation profile of the Δ*tolRA* mutant (Figure 1C). The Δ*ihfABpmi* could invade and survive inside tumor cells, but not as efficiently as the wild type (Figures 2A, B). Despite its decreased survivability, the Δ*ihfABpmi* mutant efficiently decreased the viability of tumor cells *in vivo* and *in vitro* (Figures 2C, D). Mutations in the *ihfA*, *ihfB,* and *pmi* genes did not compromise the antitumor capacity of *S. enterica* Typhimurium, as treatment with the triple mutant led to complete regression of B16F10 tumors (Figure 4) without harmful effects on normal organs (Figure 5). Several studies have shown that *S. enterica* Typhimurium exerts its antitumor activity by activating the immune system [11, 17, 35], thus the immunogenicity of the bacterium is the key to eliminating tumors.

After a few replication cycles, the inability to produce full LPS in the Δ*pmi* mutant allows the mutant to activate the immune system and acquire an additional attenuation phenotype through mannose deficiency [67]. A closer examination of the antitumor effect showed that treatment of tumors with the Δ*ihfABpmi* mutant leads to accumulation of macrophages of phenotype M1 and decrease of macrophages of phenotype M2. We found that M2-type macrophages were more abundant in mice from the control group (PBS) tumor tissue than in the Δ*ihfABpmi*-treated group. In contrast, the percentage of macrophages and M1-type macrophages was higher in the mutant-treated group (Figure 6). Our data do not allow us to know whether the accumulation M1-type macrophages and decrease of M2-type macrophages observed in tumors of mice treated with Δ*ihfABpmi* are due to Δ*ihfABpmi* induces the reprogramming of intratumoral macrophages from pro-tumor phenotype to anti-tumor phenotype or whether Δ*ihfABpmi* induces the infiltration of anti-tumor macrophages in antigenically poor tumors. Further studies are needed to elucidate this finding. Accumulation of M1-type macrophages was also accompanied with a significant increase in the mRNA of inflammatory cytokines (TNF-α and IL-6), iNOS, and Bax (Figure 7). In addition, the cytokines TNF-α, IFN-γ, IL-6, and IL-12P70 have been associated with antitumor activity in previous studies [33, 71]. Together, our results suggest that the Δ*ihfABpmi* mutant induces accumulation of M1-type macrophages, which leads to apoptosis and suppresses tumor growth by secreting cytokines with antitumor activity.

Macrophages have been demonstrated to be activated by LPS and flagellin to specifically kill tumor cells [11, 16]. LPS and flagellin present in *S. enterica* Typhimurium can stimulate the infiltration of immune cells, such as macrophages, by TLR5 and TLR4 signaling, respectively. Immune cells produce pro-inflammatory mediators such as TNF-α, IL-6, IL-1, IL-12, and iNOS, leading to reprogramming the immunosuppressive tumor microenvironment into an immunogenic one to aid in the elimination of tumors [35]. At the time of inoculation, the Δi*hfABpmi* mutant has complete LPS, allowing the bacterium to colonize efficiently and leading to reprogramming of the tumor microenvironment, evidenced by the percentage of M1 macrophages and the significant increase in iNOS, an indicator of type M1 macrophages. Our results are consistent with previous reports in models of melanoma, lymphoma, colon adenocarcinoma, and breast cancer that also show that *S. enterica* Typhimurium can induce the accumulation of macrophage phenotype to the M1 phenotype and induce the secretion of inflammatory cytokines in tumors [7, 11, 16, 35, 72].

TNF-α production is associated with apoptosis-induced tumor cell death [72, 73]. Previous studies have shown that *S. enterica* Typhimurium has an apoptotic effect by increasing Bax production [7, 74]. The data presented here show that treatment with the Δ*ihfABpmi* mutant significantly increases the expression of genes involved in the apoptosis pathway, suggesting that *S. enterica* Typhimurium exerts its antitumor activity by activating apoptosis.

Bacterial toxicity is a barrier to their use in cancer immunotherapy. The triple mutant explored in this study loses complete LPS after a few generations, which makes the bacterium more susceptible to the immune system and can be eliminated from the host without causing serious toxicity. At the endpoint of the experiment, we did not detect the Δ*ihfABpmi* mutant in the blood, tumor, liver, or spleen of mice, suggesting that our mutant reduced the tumor mass and was subsequently eliminated by the immune system. Furthermore, mutations in the genes coding for the IHF protein reduce the risk that the Δ*ihfABpmi* mutant makes homologous recombination with microbiota bacteria or that random mutations will appear. In addition, IHF mutants are further attenuated as discussed previously. It is important to mention that *S. enterica* mutants for HU, another NAP, were also attenuated and induced a protective immunological response in the murine model [29]. Altogether, these data indicate that NAP proteins represent a new target for the development of *S. enterica* attenuated strains with vaccine and antitumor potential.

In the present study, we report two attenuated mutants of *S. enterica* Typhimurium and explored their antitumor activity *in vitro* and *in vivo*. The first mutant was shown to be highly attenuated, but with compromised antitumor activity. The second mutant, *ΔihfABpmi* showed a lower attenuation profile but maintained its ability to invade and survive in tumor cells. Additionally, it was efficient in eliminating melanoma. Our results also suggest that the antitumor effect of treatment with the *ΔihfABpmi* mutant induces the accumulation of macrophages from M1-type macrophages, that secrete pro-inflammatory mediators, leading to the apoptosis of tumor cells. Taken together, our data provide a basis for understanding the activity of *S. enterica* Typhimurium in cancer immunotherapy.

## Data Availability

The experimental data that support the findings of this study are available in: https://doi.org/10.6084/m9.figshare.25962253.
