# Supplementary material for: Attenuated mutants of Salmonella enterica Typhimurium mediate melanoma regression via an immune response
Source: Exp Biol Med (Maywood). 2024 Jun 21;249:10081. doi: 10.3389/ebm.2024.10081 (PMC11224151; doi:10.3389/ebm.2024.10081)
Supplement: Supplementary file 1 [file DataSheet1.PDF]

Supplemental tables

Supplemental table 1. Strains, plasmids, and primers used to construct the mutants

**Strains, plasmids, and primers**	**Description**	**Source**
**Strains**		
14028	ATCC 14028 wild type	(Jarvik *et al.*, 2010)
Δ*ihfAB*	Δ*ihfA* Δ*ihfB*: *cat* mutant of *S.* Typhimurium ATCC 14028	Lab collection
Δ*pmi*	Δ*pmi: Kan* mutant of *S.* Typhimurium ATCC 14028	This study
Δ*ihfABpmi*	Δ*ihfA* Δ*ihfB*: *cat*Δ*pmi: Kan* mutant of *S.* Typhimurium ATCC 14028	This study
Δ*tolRA*	Δ*tolR*Δ*tolA*: *Kan* mutant of *S.* Typhimurium ATCC 14028	This study
Pseudomonas aeruginosa (Δ*wzz*)	Δ*wzz* mutant of *Pseudomonas aeruginosa*	Regina Lúcia Baldini, University of São Paulo
**Plasmids**		
pKD4	Ampr, Kanr, λRed system accessory for Kan amplification	(Datsenko & Wanner, 2000)
pKD46	Ampr, expressa γ, β e exo, λRed system accessory	(Datsenko & Wanner, 2000)
**Primers**		
*pmi*P1	5=-TTA CGT CTG TTA TAA GCT TAG CAA GAG TTG TTA AAA AAT TCA GTA CGT TGA TGG GAA T-3=	This study
*pmi*P2	5=-TCT TGG TTT AAT ACC TCC CAT TGA TCT CCA CAT TGA AAC AGG GCT TGA TAG TGT AGG-3=	This study
*pmi*DT-F	5=-CCC GAC TCA AAG CGA GTA AC-3=	This study
*pmi*DT-R	5=-ATT AAA CCG CCT GGA GGA GT-3=	This study
*tolRA*P1	5=-TGC ACC GCC AGG CGT TTA CCG TAA GCG AAA GCA ACA AGG GGT AAG CCA TGA TTC CGG GGA TCC GTC GAC C-3=	(Daleke-Schermerhorn *et al.*, 2014)
*tolRA*P2	5=-ACT GCT CTA ACT TCC ATA AAG AAA AGT ATC TAC AGT TTA AAG TCT AGT TTG GCT GTA GGC TGG AGC TGC TTC G-3=	(Daleke-Schermerhorn *et al.*, 2014)
*tolRA*DT-F	5=-CGTAAGCGAAAGCAACAAGG-3=	(Daleke-Schermerhorn *et al.*, 2014)
*tolRA*DT-R	5=-CCACCAGGACCAGTA ACA AC-3=	(Daleke-Schermerhorn *et al.*, 2014)

Supplemental table 2. Primers used for qRT-PCR.

Target gene	Amplicon size	Primers sequence (5’ – 3′)
β-actin	154 pb	F:GGCTGTATTCCCCTCCATCG<br>R:CCAGTTGGTAACAATGCCATGT
GAPDH	133 pb	F: GCGAGACCCCACTAACATCA<br>R: GGCGGAGATGATGACCCTTT
TNF-α	139 pb	F:GGTGCCTATGTCTCAGCCTCTT<br>R:GCCATAGAACTGATGAGAGGGAG
IL-6	129 pb	F: ACGGCCTTCCCTACTTCACA<br>R:CATTTCCACGATTTCCCAGA
Ki-67	104 pb	F: CCTTTGCTGTCCCCGAAGA<br>R: GGCTTCTCATCTGTTGCTTCCT
VEGF	105 pb	F: GCACATAGAGAGAATGAGCTTCC<br>R:CTCCGCTCTGAACAAGGCT
Bax	187 pb	F:AGGCCTCCTCTCCTACTTCG<br>R:AAATGCCTTTCCCCTTCCCC
INOS	127 pb	F:GTTCTCAGCCCAACAATACAAGA<br>R:GTGGACGGGTCGATGTCAC

Supplemental Figures

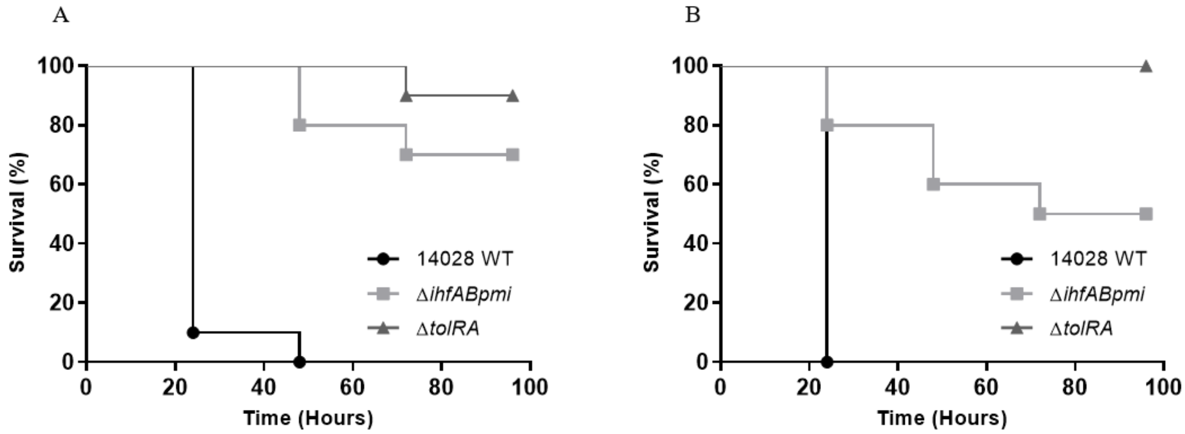

Supplemental Figure 1. Independent experiments of the *Galleria mellonella* infection model used to assess attenuation of *S. enterica* Typhimurium mutants. Larvae were inoculated with 10^4 CFU in the last proleg, and survival was observed for 96 hours.

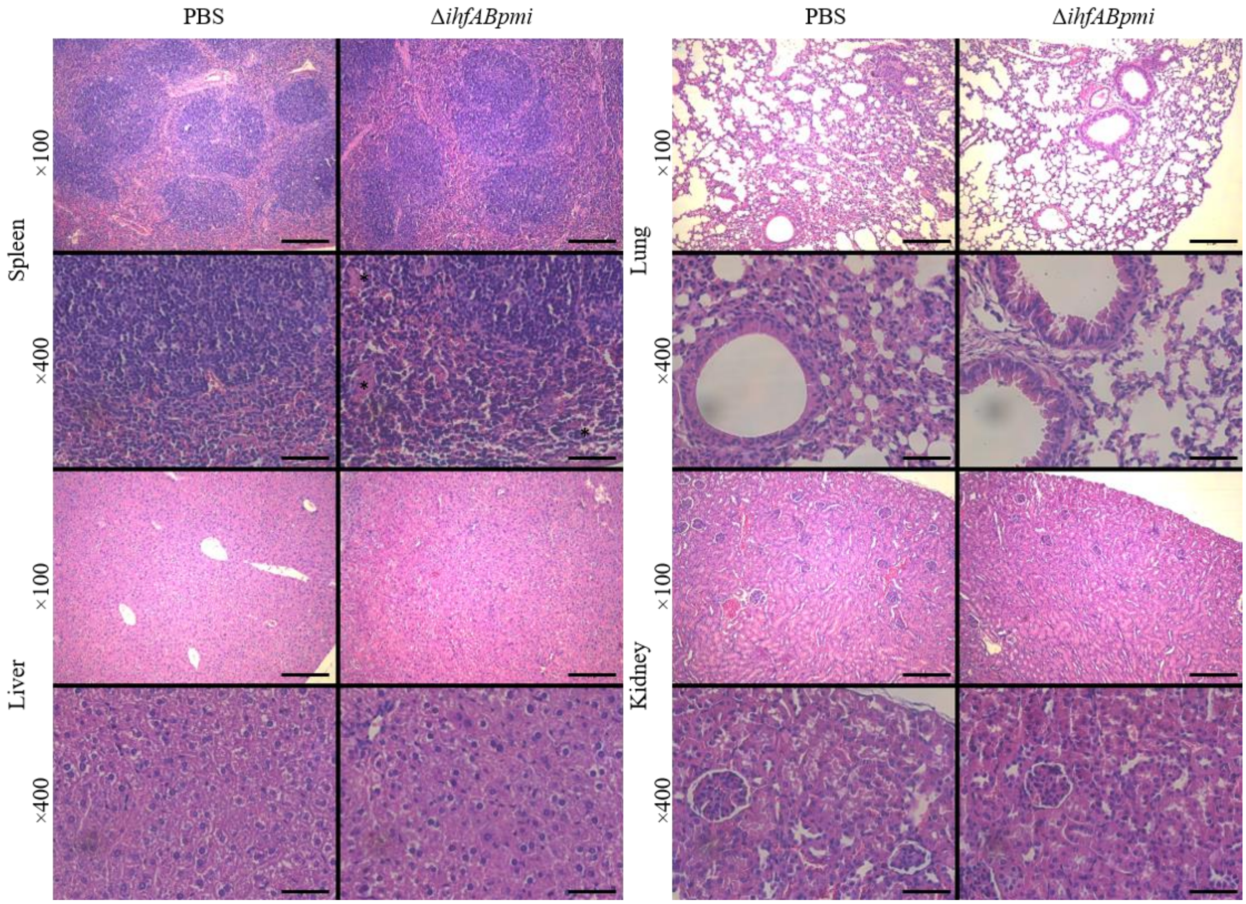

Supplemental Figure 2. Safety of treatment with Δ*ihfABpmi* mutant in normal organs. Healthy C57BL/6junib mice were inoculated with 10^5 UFC of Δ*ihfABpmi* mutant or PBS twice a week for two weeks. After one week of the last inoculation, the spleen, liver, lung, and kidney were collected for histological analysis. H&E staining of organ sections. The asterisks indicate the megakaryocytes in the spleens. Scale bar: 200 μm for ×100 and 50 μm for ×400.
